# Glutamate-induced apoptosis in primary cortical neurons is inhibited by equine estrogens via down-regulation of caspase-3 and prevention of mitochondrial cytochrome c release

**DOI:** 10.1186/1471-2202-6-13

**Published:** 2005-02-24

**Authors:** YueMei Zhang, Bhagu R Bhavnani

**Affiliations:** 1 Department of Obstetrics and Gynecology, University of Toronto, Toronto, Canada; 2 Institute of Medical Sciences, University of Toronto, Toronto, Canada; 3 Department of Obstetrics and Gynecology, St. Michael's Hospital, Toronto, Canada

## Abstract

**Background:**

Apoptosis plays a key role in cell death observed in neurodegenerative diseases marked by a progressive loss of neurons as seen in Alzheimer's disease. Although the exact cause of apoptosis is not known, a number of factors such as free radicals, insufficient levels of nerve growth factors and excessive levels of glutamate have been implicated. We and others, have previously reported that in a stable HT22 neuronal cell line, glutamate induces apoptosis as indicated by DNA fragmentation and up- and down-regulation of Bax (pro-apoptotic), and Bcl-2 (anti-apoptotic) genes respectively. Furthermore, these changes were reversed/inhibited by estrogens. Several lines of evidence also indicate that a family of cysteine proteases (caspases) appear to play a critical role in neuronal apoptosis. The purpose of the present study is to determine in primary cultures of cortical cells, if glutamate-induced neuronal apoptosis and its inhibition by estrogens involve changes in caspase-3 protease and whether this process is mediated by Fas receptor and/or mitochondrial signal transduction pathways involving release of cytochrome c.

**Results:**

In primary cultures of rat cortical cells, glutamate induced apoptosis that was associated with enhanced DNA fragmentation, morphological changes, and up-regulation of pro-caspase-3. Exposure of cortical cells to glutamate resulted in a time-dependent cell death and an increase in caspase-3 protein levels. Although the increase in caspase-3 levels was evident after 3 h, cell death was only significantly increased after 6 h. Treatment of cells for 6 h with 1 to 20 mM glutamate resulted in a 35 to 45% cell death that was associated with a 45 to 65% increase in the expression of caspase-3 protein. Pretreatment with caspase-3-protease inhibitor z-DEVD or pan-caspase inhibitor z-VAD significantly decreased glutamate-induced cell death of cortical cells. Exposure of cells to glutamate for 6 h in the presence or absence of 17β-estradiol or Δ^8^, 17β-estradiol (10 nM-10 μM) resulted in the prevention of cell death and was associated with a significant dose-dependent decrease in caspase-3 protein levels, with Δ^8^, 17β-E_2 _being more potent than 17β-E_2_. Protein levels of Fas receptor remained unchanged in the presence of glutamate. In contrast, treatment with glutamate induced, in a time-dependent manner, the release of cytochrome c into the cytosol. Cytosolic cytochrome c increased as early as 1.5 h after glutamate treatment and these levels were 5 fold higher after 6 h, compared to levels in the untreated cells. Concomitant with these changes, the levels of cytochrome c in mitochondria decreased significantly. Both 17β-E_2 _and Δ^8^, 17β-E_2 _reduced the release of cytochrome c from mitochondria into the cytosol and this decrease in cytosolic cytochrome c was associated with inhibition of glutamate-induced cell death.

**Conclusion:**

In the primary cortical cells, glutamate-induced apoptosis is accompanied by up-regulation of caspase-3 and its activity is blocked by caspase protease inhibitors. These effects of glutamate on caspase-3 appear to be independent of changes in Fas receptor, but are associated with the rapid release of mitochondrial cytochrome c, which precedes changes in caspase-3 protein levels leading to apoptotic cell death. This process was differentially inhibited by estrogens with the novel equine estrogen Δ^8^, 17β-E_2 _being more potent than 17β-E_2_. To our knowledge, this is the first study to demonstrate that equine estrogens can prevent glutamate-induced translocation of cytochrome c from mitochondria to cytosol in rat primary cortical cells.

## Background

High concentrations (mM) of the excitatory neurotransmitter glutamate can accumulate in the brain and are thought to be involved in the etiology of a number of neurodegenerative disorders including Alzheimer's disease [1-4]. A number of *in vitro *studies indicate that at high concentrations, glutamate is a potent neurotoxin capable of destroying neurons [5,6].

The mechanisms by which glutamate-induced neurotoxicity or excitotoxicity is mediated, has not been established, however, a substantial body of evidence suggests that glutamate toxicity involves oxidative stress and apoptosis (programmed cell death) [2,7-9]. This latter form of cell death is characterized by DNA degradation that results by cleaving DNA at internucleosomal sites [10]. Apoptosis is a gene-directed process and an increasing number of genes and their proteins are involved in this process [11,12]. We have previously reported that in a stable mouse hippocampal neuronal cell line (HT22), glutamate-induced cell death is associated with DNA fragmentation and up-regulation of the pro-apoptotic protein Bax and down-regulation of the anti-apoptotic protein Bcl-2, however, in this cell line, the apoptotic process did not appear to involve caspase-3 [13]. In contrast, recent studies demonstrate that a family of cysteine proteases (caspases) play an important role in apoptotic cell death observed in some neurodegenerative diseases [14-16]. Caspase-3 is considered to be the central and final apoptotic effector enzyme responsible for many of the biological and morphological features of apoptosis [15-17]. Caspase-3 usually exists in the cytosolic fraction of cells as an inactive precursor that is activated proteolytically by cleavage at a specific amino acid sequence to form the active enzyme [18] which is capable of cleaving several proteins that culminate in apoptotic cell death [19]. Although these observations strongly indicate that caspase-3 is essential for apoptosis in mammalian cells, the mechanisms involved in caspase-3 regulation of the neuronal system remain to be elucidated. Many signal transduction pathways such as Fas receptor-mediated signaling pathway via caspase-8, via activation of granzyme B, or the damage of mitochondria that results in cytochrome c release, have been implicated in the initiation of caspase-3 cascade [20-25].

A number of studies have demonstrated that estrogens are potent antioxidants capable of inhibiting some of the neurotoxic effects of oxidative stress [7,26,27]. We, and others have shown that estrogens can increase cell survival and attenuate *in vitro *cell death induced by various neurotoxins [28-31]. We have also previously demonstrated in HT22 cells and in the neuronal-like PC12 cell line derived from rat adrenal pheochromocytoma cells that the neurotoxic effects of glutamate can be inhibited differentially by various equine estrogens [31]. The data further indicated that the less estrogenic (uterotropic) Δ^8^-estrogens were the most effective neuroprotectors [31]. We further suggested that the increased potency of these Δ^8^-estrogens may to some extent, be due to their greater antioxidant properties [28,29,31]. However, the mechanism(s) involved in estrogen mediated neuroprotection are not fully understood. In the present study, we have determined whether glutamate-induced neuronal apoptosis in primary cortical cells and its inhibition by estrogen, involves changes in caspase-3 protease and whether this process is mediated by Fas receptor and/or mitochondrial signal transduction pathways involving release of cytochrome c. The estrogens selected for this study were 17β-estradiol (17β-E_2_) which has high affinity for both estrogen receptors ERα and ERβ and Δ^8^, 17β-estradiol (Δ^8^, 17β-E_2_), an estrogen that is a more potent antioxidant than 17β-E_2 _and whose activity appears to be mediated to a greater extent via ERβ [31-34].

## Results

### Effects of various concentrations of glutamate on cortical cell viability

**Figure 1:**
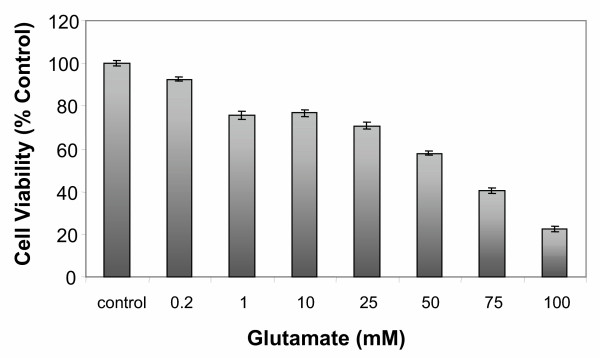
**Effects of various concentrations of glutamate on cell viability. **Primary cortical cells isolated from 18–20 day old fetal rat brains were cultured for 7 days and then treated with various concentrations of glutamate or in absence of glutamate (control) for 18 h. Cell viability was determined using the MTS cell proliferation assay as described under "Methods". Cell viability is expressed as the mean (± SEM) from three experiments. * P < 0.05 (1-100 mM)compared with untreated control cells.

Cortical cells (2.5 × 10^4^) cultured for 7 days in 96-well plates were treated with increasing concentrations (0.2 – 100 mM) of glutamate for 18 h. As depicted in Figure 1, increasing concentrations of glutamate resulted in a dose-dependent decrease in cell viability measured by the MTS (3-[4,5-dimethylthiazol-2 yl]-5 [3 carboxymethoxyphenyl] 2 H-tetrazolium, inner salt) cell proliferation assay as described under "Methods". Generally as the amount of glutamate increased, cell death increased progressively, however, there was no significant difference between 1 and 25 mM glutamate in 18 h. Similar results were obtained after treatment with glutamate for 6 h (data not shown). Based on these preliminary data, all subsequent experiments were carried out for 6 h at glutamate concentrations between 1 to 20 mM. At these concentrations, the mean percent cell death was approximately 20%.

### Demonstration of apoptosis in cortical cells treated with glutamate

**Figure 2:**
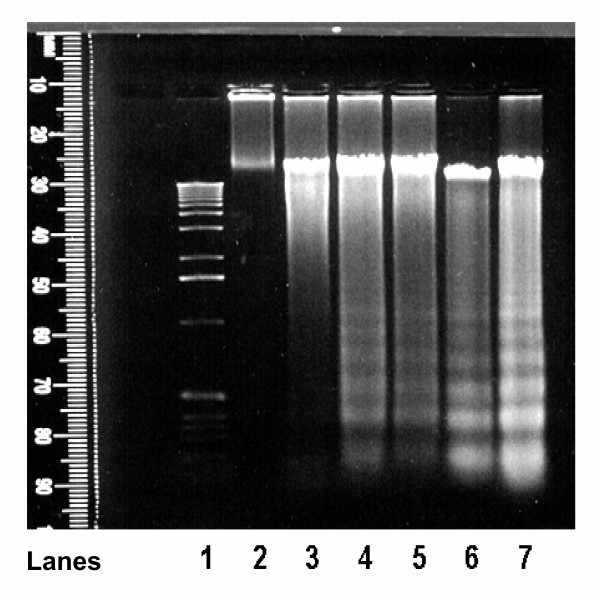
**Detection of glutamate induced DNA fragmentation (DNA ladder) **Primary cortical cells isolated from 18–20 day old fetal rat brains were cultured for 7 days and treated with 1 mM glutamate for 18 h. The cells were processed as described under "Methods". The isolated DNA was electrophoresed on TBE agarose gel for 1.5 h at 100 V. The DNA fragments were visualized by staining with ethidium bromide. Lane 1 = 1 kb DNA ladder; lane 2 = DNA from untreated cortical cells prior to culture; lane 3 = DNA from untreated control cells cultured for 1 day; lane 4 = DNA from untreated control cells cultured for 2 days; lane 5 = DNA from cells cultured for 2 days and treated with glutamate for 18 h; lane 6 = DNA from untreated control cells cultured for 7 days; lane 7 = DNA from cells cultured for 7 days and then treated for 18 h with glutamate. Note the potentiation of DNA fragmentation in presence of glutamate.

**Figure 3:**
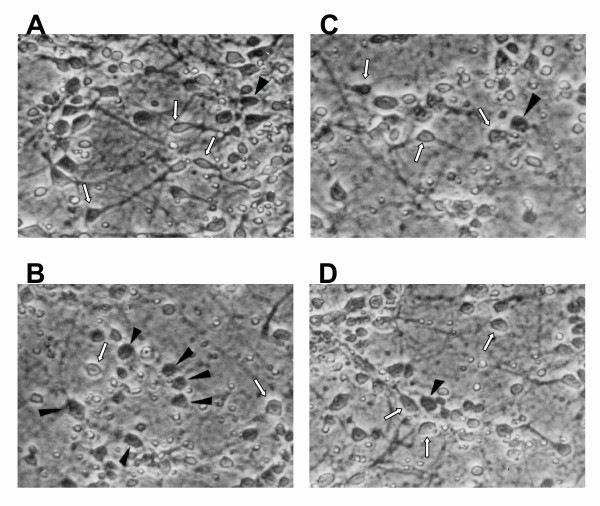
**Glutamate-induced toxicity in rat primary cortical cells as assessed by phase contrast microscopy (100×). **Primary cortical cells isolated from 19 day old fetal rat brains were cultured for 7 days and then treated with 1 mM glutamate alone or in the presence of estrogens for 6 h. Degenerated cells are depicted by arrowheads. A = untreated control cells; B = cells treated with 1 mM glutamate for 6 h; C = cells treated with 1 mM glutamate and 1 μM 17β-E_2_; D = cells treated with 1 mM glutamate and 1 μM Δ^8^, 17β-E_2_. Note after glutamate treatment, dead cells with loss of dendrites are clearly visible in the cultures. In contrast, either untreated control cells or cells treated with glutamate in presence of estrogens retain their normal morphology. Dendrites and membranes are clearly visible and only a few degenerated cells are present in these cultures (A,C,D).

Cortical cells cultured in poly-lysine coated 6-well plates for up to 7 days were treated with 1 mM glutamate for 18 h. DNA was extracted, purified and subjected to agarose gel electrophoresis as described under "Methods". The results indicate (Figure 2), that in DNA isolated from untreated cortical cells prior to culture (Figure 2, lane 2), untreated cells in culture for 1 day (Figure 2, lane 3) no DNA fragmentation was detectable. However, after 2 and 7 days in culture, DNA fragmentation was detectable (Figure 2, lanes 4 and 6). The extent of DNA fragmentation was potentiated in cells treated for 18 h with 1 mM glutamate (Figure 2, lanes 5 and 7). These results indicate that cortical cells in culture for longer than 2 days display characteristic DNA fragmentation or laddering that is associated with apoptosis mediated via caspase-3 activation. Glutamate treatment as can be seen in Figure 2, lane 7 induced a much greater DNA fragmentation than untreated cortical cells. However, these are qualitative data and should be interpreted with caution, particularly since some DNA fragmentation occurs in untreated cells. Glutamate-induced enhancement of apoptosis was also detectable by characteristic morphological changes observed by using phase contrast microscopy (Figure 3). After 6 h in culture, untreated cortical cells retained normal morphology of neuronal cortical cells and their cellular extensions (dendrites) and membranes were clearly visible. An occasional degenerated cell was also visible (Figure 3A). In contrast, after a 6 h incubation with 1 mM glutamate degenerated, dead or apoptotic cells were clearly visible in these cultures (Figure 3B), and the cellular extensions seen in untreated cortical cells (Figure 3A), were retracted, and cells appeared rounded. Similar changes were also observed with 5 mM glutamate (data not shown). In the presence of 1 mM glutamate and 1 μM, 17β-E_2 _(Figure 3C) and Δ^8^, 17β-E_2 _(Figure 3D), the cells retained the normal morphology of untreated cells, and only an occasional degenerated cell was visible. These results clearly confirm that glutamate induces and enhances apoptosis. These morphological changes and cell death were prevented by both estrogens. Taken together, these data indicate that cortical cells in the presence of glutamate undergo apoptotic changes in culture.

### Effects of glutamate and estrogens on cell death

**Figure 4:**
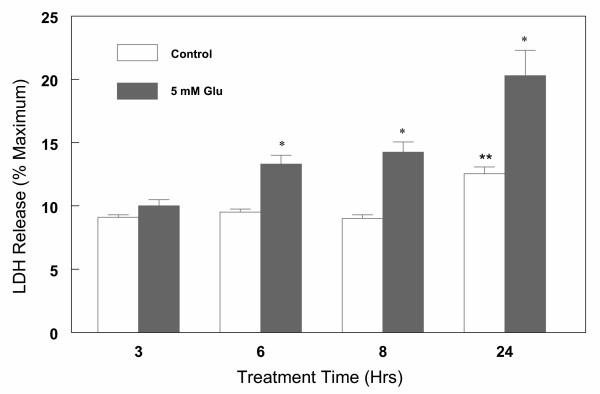
**Kinetics of glutamate-induced cell death in primary cortical cells. **Cortical cells isolated from 19 day old fetal rat brains were cultured for 7 days and treated with 5 mM glutamate for the indicated times in a neurobasal medium containing 2% antioxidant free B27. LDH release (% maximum) was measured after 3,6,8 and 24 h of treatment. Data are the mean ± SEM values from at least 3 experiments. *P < 0.05 compared with control ** P < 0.05 compared to 3,6,8 h control.

**Figure 5:**
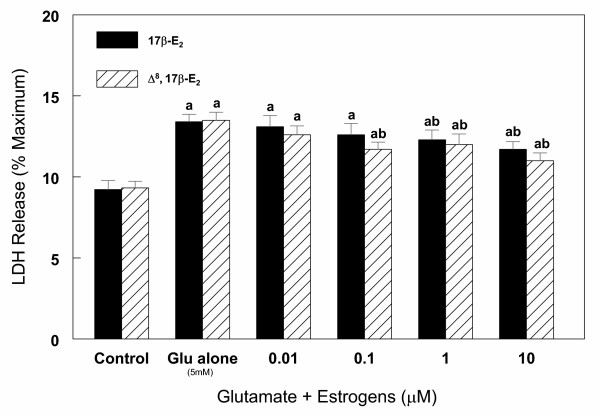
**Effect of various concentrations of 17β-E_2 _and Δ^8^, 17β-E_2 _on glutamate (5 mM) induced cell death in primary cortical cells. **Cortical cells isolated from 19 day old fetal rat brains were cultured for 7 days and treated with 5 mM glutamate alone or in the presence of estrogens in neurobasal medium containing 2% antioxidant free B27. LDH release (% maximum) was measured after 6 h treatment. Data are the mean ± SEM values from at least 3 experiments. a = P < 0.05 compared with untreated control cells; b = P < 0.05 compared with glutamate alone.

Cortical cells were cultured in 6-well plates (1 × 10^6 ^cells/well) for 7 days. The medium was then changed and the effects of various concentrations (1–20 mM) of glutamate on lactate dehydrogenase (LDH) release were measured for 6 h. The results indicate that glutamate induced a significant (30–40%) increase in LDH release compared to the control untreated cells. No significant difference in LDH release was observed in 6 h at glutamate concentration between 1 to 20 mM, (data not shown). Based on these observations and the cell viability data (Figure 1), all subsequent experiments were done in the presence of 1 or 5 mM glutamate to avoid potential necrosis which may occur at higher concentration of glutamate. In order to follow the kinetics of glutamate cytotoxicity, the effect of 5 mM glutamate on LDH release as a function of time was measured for up to 24 h. The results shown in Figure 4 indicate that glutamate toxicity varied markedly during the course of culture. Significant increase (40–60%) in cytotoxicity was observed after 6 to 24 h exposure of cells to glutamate. During this period, LDH release in untreated control cells remained the same for up to 8 h and significantly increased (P < 0.05) after 24 h (Figure 4). Next, cortical cells were treated with 5 mM glutamate for 6 h in the presence or absence of various concentrations (0.01–10 μM) of 17β-E_2 _and Δ^8^, 17β-E_2_. Release of LDH from cells was measured and the results are summarized in Figure 5. Both 17β-E_2 _and Δ^8^, 17β-E_2 _inhibited glutamate-induced cell death in a dose-dependent manner, with Δ^8^, 17β-E_2 _being more potent. Thus, 0.1 μM Δ^8^, 17β-E_2 _and 1 μM 17β-E_2 _significantly reduced cell death compared to glutamate alone (Figure 5). However, even at the highest concentration of estrogens tested, cell death induced by 5 mM glutamate was not fully preventable. In contrast, when cell death was induced by lower concentration (1 mM) of glutamate, 10 μM of Δ^8^, 17β-E_2 _completely inhibited cell death and the release of LDH returned to control levels (data not shown).

### Effects of glutamate and estrogens on caspase-3 protein levels

**Figure 6:**
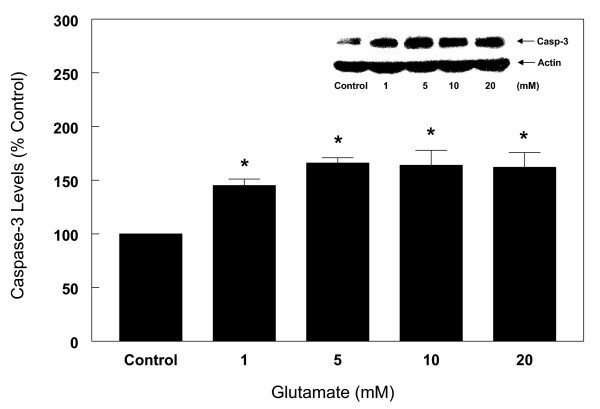
**Effects of various concentrations of glutamate on caspase-3 protein levels in primary cortical cells. **Cortical cells isolated from 19 day old fetal rat brains were cultured for 7 days and treated with 1,5,10 and 20 mM glutamate for 6 h in a neurobasal medium containing 2% antioxidant free B27. Cells were then harvested and total protein was extracted. Caspase-3 protein levels were assessed by Western blot analysis as described under "Methods". Actin was used as loading control. The bars depict densitometric analyses of Western blots from at least three experiments (± SEM). *P < 0.05 compared with untreated control cells.

**Figure 7:**
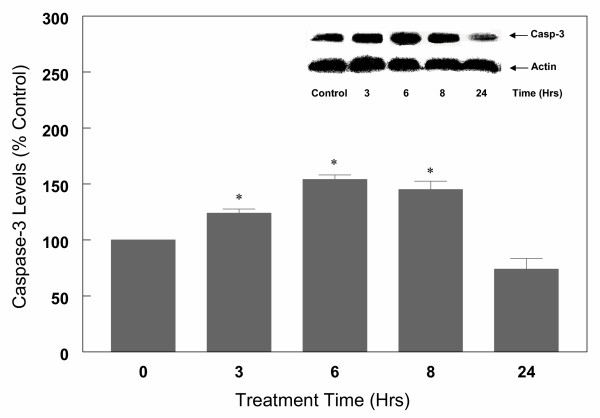
**Kinetics of glutamate (5 mM) treatment on caspase-3 protein levels in primary cortical cells. **Cortical cells isolated from 19 day old fetal rat brains were cultured for 7 days and treated with 5 mM glutamate for the indicated times in a neurobasal medium containing 2% antioxidant free B27. Cells were then collected and total protein was extracted. Caspase-3 protein levels were determined by Western blot analysis as described in Figure 6. The bars depict densitometric analyses of Western blots from at least three experiments (± SEM). *P < 0.05 compared to untreated control cells.

**Figure 8:**
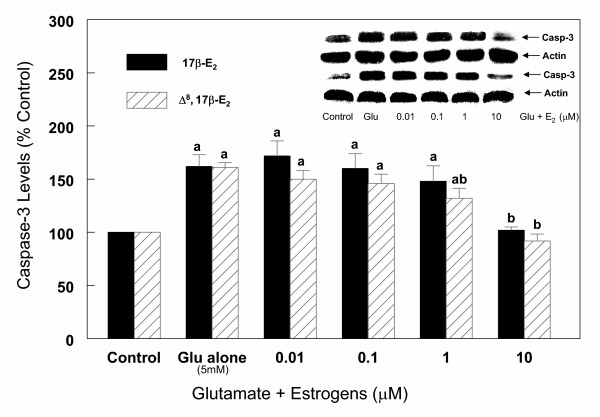
**Effects of estrogens on caspase-3 protein levels during glutamate (5 mM) induced cell death in primary cortical cells. **Cortical cells isolated from 19 day old fetal rat brains were cultured for 7 days and treated with 5 mM glutamate alone or in the presence of 17β-E_2 _and Δ^8^, 17β-E_2 _for 6 h. Cells were then harvested and total protein was extracted. Caspase-3 protein levels were determined by Western blot analysis as described under Figure 6. The bars depict densitometric analyses of Western blots from at least three experiments (± SEM). a = P < 0.05 compared with untreated control cells, b = P < 0.05 compared with glutamate alone.

**Figure 9:**
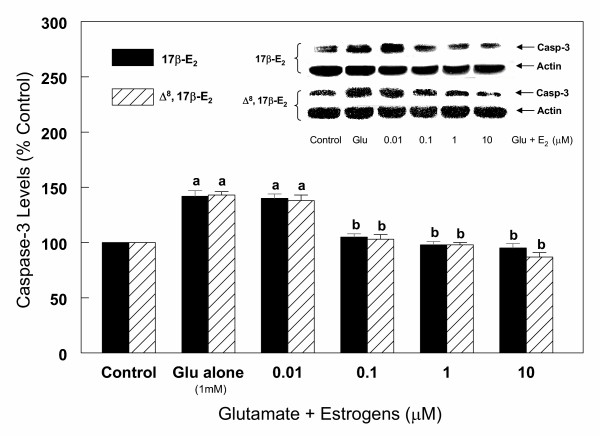
**Effects of estrogens on caspase-3 protein levels during glutamate (1 mM) induced cell death in primary cortical cells. **Cortical cells isolated from 19 day old fetal rat brains were cultured for 7 days and treated with 1 mM glutamate alone or in the presence of 17β-E_2 _and Δ^8^, 17β-E_2 _for 6 h. Cells were then harvested and total protein was extracted. Caspase-3 protein levels were determined by Western blot analysis as described under Figure 6. The bars depict densitometric analyses of Western blots from at least three experiments (± SEM). a = P < 0.05 compared with untreated control cells, b = P < 0.05 compared with glutamate alone.

Following measurement of LDH, cortical cells were harvested, lysed and processed for Western blot analysis as described under "Methods". The results (Figure 6) indicate that both anti-caspase-3 antibodies detected the presence of caspase-3 Mr 32 kDa protein band (precursor protein), but not the p 20 and p 11 active fragments of caspase-3. The results further indicate that the exposure of cells to glutamate resulted in an increase in caspase-3 precursor protein in a dose (Figure 6) and time (Figure 7), dependent manner. Thus, 1 to 5 mM glutamate increased caspase-3 protein levels by 45 to 66% respectively, however, higher concentrations of glutamate (10–20 mM) did not result in any further increase in caspase-3 protein levels (Figure 6). The kinetics of glutamate effects on caspase-3 protein levels indicate that a significant increase in the levels occurred by 3 h of glutamate (5 mM) exposure and reached maximum levels observed at 6 h (54% increase) (Figure 7). The levels were significantly lower at 24 h (Figure 7), most likely due to decreased transcription of caspase-3. These results further indicate that changes in levels of caspase-3 occur soon after induction of apoptosis. Lack of further increase in caspase-3 protein levels suggest that cell death at this late stage may be due to necrosis and therefore all subsequent experiments were carried out for up to 6 h only. These glutamate-induced changes were reversed in the presence of estrogens. Thus, increasing concentrations of 17β-E_2 _and Δ^8^, 17β-E_2 _in the presence of 5 mM glutamate for 6 h resulted in a decrease in caspase-3 levels in a dose-dependent manner (Figure 8). As depicted in Figure 8, glutamate (5 mM) alone increased the levels of caspase-3 by 60% and in the presence of 1 to 10 μM 17β-E_2_, the levels of caspase-3 protein decreased gradually and returned to control values with 10 μM 17β-E_2_. Similar results were obtained with Δ^8^, 17β-E_2_, however, significant decrease (30%) in the levels of caspase-3 occurred at 10 times lower concentration (1 μM) (Figure 8). When cell death was induced with lower concentration (1 mM) of glutamate, 0.1 μM 17β-E_2 _and Δ^8^, 17β-E_2 _completely inhibited the changes in caspase-3 protein and the levels returned to control values (Figure 9). Whether the ability of low concentrations of estrogens to fully protect the cortical cells against neurotoxicity induced by 1 mM glutamate was due to the cell death resulting via predominantly apoptosis rather than necrosis or a mixture of the two processes remains to be investigated.

### Effects of caspase inhibitors, z-DEVD and z-VAD, on glutamate-induced cell death

**Figure 10:**
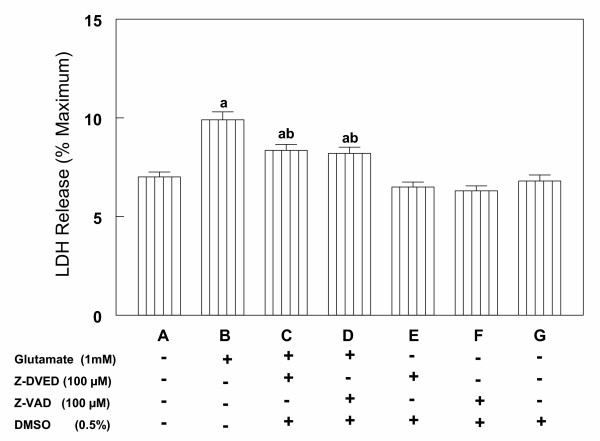
**Effects of caspase inhibitors on glutamate-induced cell death in primary cortical cells. **Cortical cells isolated from 19 day old fetal rat brains were cultured for 7 days. Cell death was measured by LDH release assay 6 h after incubation with 1 mM glutamate with or without 30 min pretreatment with 100 μM caspase-3 specific inhibitor z-DEVD or a pan-caspase inhibitor z-VAD (A,B,C and D). Inhibitors alone (E and F) or DMSO (G) had not effect on LDH release. Data are the mean ± SEM for 3 experiments performed. a = P < 0.05 compared with control, b = P < 0.05 compared with glutamate alone.

To further confirm the role of caspase-3 in glutamate-induced cell death, cortical cells were pre-incubated with 100 μM of z-DEVD or z-VAD prior to induction of cell death by 1 mM glutamate. Glutamate alone caused a significant increase in cell death over control (Figures 10 A, B), while pre-incubation with the caspase-3 inhibitors prior to glutamate exposure resulted in a 50% reduction in cell death (Figures 10 C, D). In absence of glutamate, both inhibitors had no effect on cell viability (Figures 10 E, F). Similarly, DMSO, the vehicle used in the preparation of the inhibitors had no effect on cell viability (Figure 10 G). These data confirm that in primary culture of rat cortical cells glutamate induces cell death via the apoptotic pathway that involves changes in caspase-3 protease.

### Activation of caspase-3

#### (a) Effects of caspase inhibitors on proleolytic cleavage of protein kinase c (PKC) in untreated primary cortical cells and cells treated with glutamate

**Figure 11:**
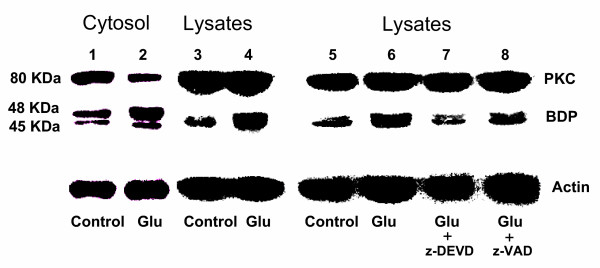
**Effect of caspase inhibitors on proteolytic cleavage of PKC in primary cortical cells treated with glutamate. **Primary cortical cells were cultured for 7 days and then treated with 1 mM glutamate with or without 30 minutes of pre-treatment with 100 μM caspase-3 specific inhibitor z-DEVD or a pan-caspase inhibitor z-VAD. After 6 h cells were harvested and total protein was extracted from the cytosol and cell lysates. PKC and its fragments were assessed by Western blot analysis as described under "Methods". Immunoblots indicate the presence of PKC (80 kDa) and its 48 kDa and 45 kDa breakdown products (BDPs). Note that glutamate treatment results in the formation of greater amounts of the 2 BDPs (lanes 2 and 4) in the cytosol and cell lysates compared to the untreated controls (lanes 1 and 3). Pre-treatment of cortical cells with 100 μM z-DEVD or z-VAD prior to glutamate inhibits the formation of PKC to its BDPs to levels seen in untreated control cells (lanes 7,8).

During apoptosis, activation of caspases including caspase-3 can result in the generation of breakdown products (BDPs) of PKC [35,36]. Primary cortical cells were either treated with glutamate alone or first pretreated with caspase-3 specific inhibitor z-DEVD or pan-caspase inhibitor z-VAD as described under "Methods". The immunoblots are depicted in Figure 11. Glutamate treatment resulted in an increase in the formation of two BDPs (48 kDa and 45 kDa) in both the cytosol and cell lysates. Pretreatment with z-DEVD and z-VAD reduced the amounts of the two BDPs to levels seen in the untreated primary cortical cells (Figure 11). These results clearly indicate that pro-caspase-3 is activated and that active caspase-3 is present in primary cortical cells and cells treated with glutamate.

#### (b) Detection of active caspase-3 in primary cortical cells treated with glutamate

**Figure 12:**
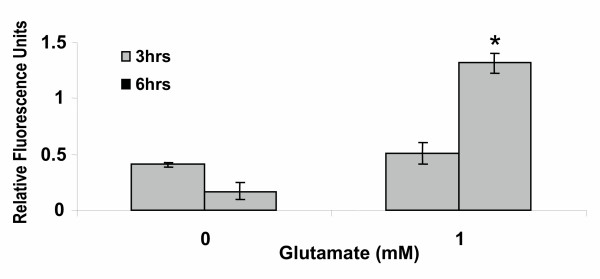
**Detection of active caspase-3 by immunofluorescence. **Primary cortical cells were cultured for 7 days in 96-well microplates and then treated with or without 1 mM glutamate for 3 h and 6 h. The cells were fixed and then incubated first with primary rabbit anti-cleaved caspase-3 antibody and then with FITC-goat anti-rabbit secondary antibody as described under "Methods". After aspiration, the cells were washed twice with PBS + saponin and the relative fluorescence measured using a Labsystem Fluroskan Accent FL microplate reader (excitation 490 nm; 520 nm emission). The bars depict relative fluorescence units from 3 measurements (± SEM). P < 0.05 compared to control untreated cells.

Western blot analysis had indicated the presence of pro-caspase-3 in primary cortical cells treated with glutamate (Figure 12). To detect the presence of active caspase subunits, highly specific active caspase-3 antibody raised against amino acids 163 to 175 (p 18 subunit) of murine caspase-3 was obtained from Stratagene. Primary cortical cells were treated with 1 mM glutamate for 3 and 6 h and processed as described under "Methods". The results (Figure 12) indicate that glutamate treatment for 3 h, did not significantly change the levels of caspase-3 active form. However, as can be seen (Figure 12) after 6 h of glutamate treatment, the levels of caspase-3 active form increased over 2 fold compared to untreated cells (Figure 12). These results further confirm that caspase-3 is activated in apoptosis induced by glutamate in primary cortical cells.

### Effects of glutamate on Fas receptor protein expression

**Figure 13:**
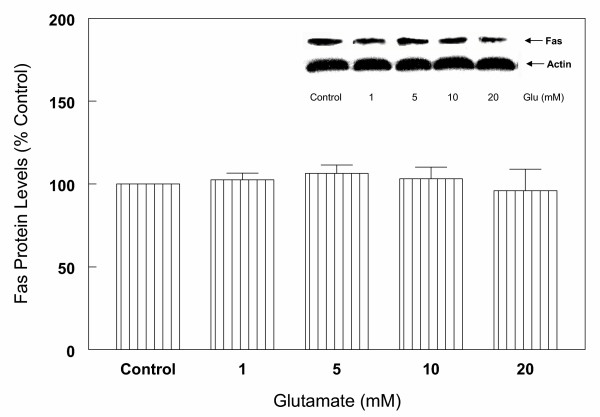
**Effects of various concentration of glutamate on Fas receptor protein levels in primary cortical cells. **Cortical cells isolated from 19 day old fetal rat brains were cultured for 7 days and treated with 1,5,10 and 20 mM glutamate for 6 h. Cells were harvested and total protein was extracted. Fas receptor protein levels were assessed by Western blot analysis as described under Figure 6. The results show that glutamate had no significant effect on Fas receptors. Data are the mean (± SEM) from 3 experiments.

**Figure 14:**
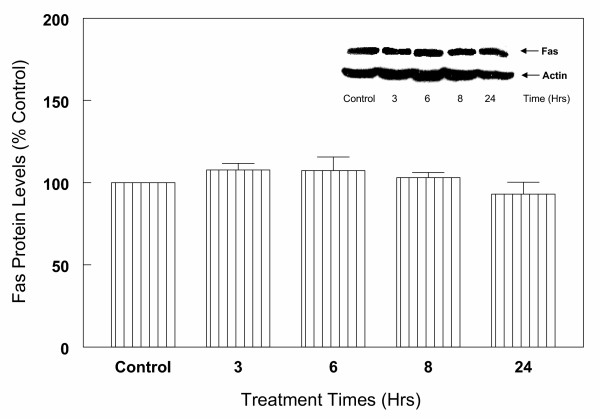
**Kinetics of glutamate (5 mM) treatment on Fas protein levels in primary cortical cells. **Cortical cells isolated from 19 day old fetal rat brains were cultured for 7 days and treated with 5 mM glutamate for the indicated times. Cells were collected and total protein was extracted. Fas receptor protein levels were determined by Western blot analysis as described under Figure 6. The results show that glutamate has no significant effects on Fas receptors up to 24 h of treatment. Data are the mean (± SEM) from 3 experiments.

To determine whether the regulation of caspase-3 in glutamate-induced cell death in cortical cells is modulated by Fas receptor mediated apoptotic pathway, expression of Fas receptor protein was evaluated by Western blot analysis. The results indicate (Figure 13) that anti-Fas receptor antibody reacted specifically with Fas receptor protein, however, no significant changes in the levels of this protein were observed following treatment with various concentrations (1–20 mM) of glutamate (Figure 13) for up to 24 hours (Figure 14) compared to control levels.

### Effects of glutamate and estrogen on the release of mitochondrial cytochrome c into the cytosol

**Figure 15:**
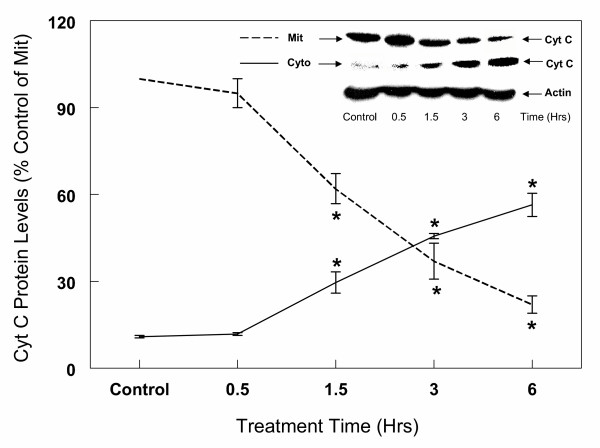
**Kinetics of glutamate (1 mM) induced Cyto c release from mitochondria into cytosol in primary cortical cells. **Primary cortical cells isolated from 19 day old fetal rat brains were cultured for 7 days and then treated with 1 mM glutamate for the indicated times. Mitochondrial and cytosol fractions were isolated as described in "Methods". Protein levels of Cyto c in mitochondria and cytosol were determined by Western Blot analysis as described under Figure 6. Actin bands were also used to monitor the same blot to verify consistency of cytosolic protein loading. The content of Cyto c was measured by densitometric scanning of the film. The results are the mean (± SEM) of at least three experiments. *P < 0.05 compared with control.

**Figure 16:**
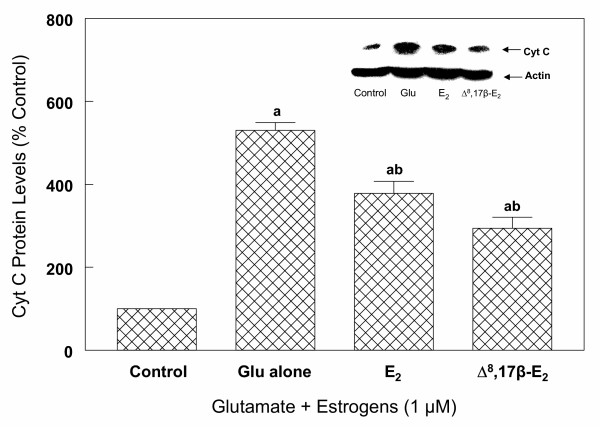
**Effects of 17β-E_2 _and Δ^8^, 17β-E_2 _on glutamate-induced Cyto c release into cytosol in primary cortical cells. **Primary cortical cells isolated from 19 day old fetal rat brains were cultured for 7 days and treated with 1 mM glutamate alone or in the presence of 17β-E_2 _and Δ^8^, 17β-E_2_. After 6 h, cells were collected and cytosol fraction was prepared as described under "Methods". Cytosolic protein levels of Cyto c were determined by Western blot analysis as described under Figure 6. Actin bands were used to monitor the same blot to verify consistency of protein loading. The content of Cyto c was measured by densitometric scanning of the film. The results are the mean (± SEM) of at least three experiments. a = P < 0.05 compared with control; b = P < 0.05 compared with glutamate treated cells.

To determine whether the activation of caspase-3 in glutamate-induced cell death requires cytochrome c, primary cultures of cortical cells were first incubated with 1 mM glutamate for up to 6 hours. The cells were then processed for Western blot analysis as described under "Methods". The results depicted in Figure 15 clearly indicate that prior to glutamate treatment, the bulk of cytochrome c is localized in the mitochondria and barely detectable levels were observed in the cytosol. In contrast, exposure of cells to glutamate resulted in a rapid release of cytochrome c from the mitochondria into the cytosol (Figure 15). Significant increase in cytosolic cytochrome c occurred as early as 1.5 h, and after 3 h, the levels of cytochrome c were higher in the cytosol compared to the mitochondria (Figure 15). By 6 h, the levels of cytochrome c in the cytosol were 2.5 times higher than those in the mitochondria. The cytosolic levels of cytochrome c between 1.5 hours to 6 hours were 2 to 5 fold higher in the cytosol prepared from glutamate treated cells compared to untreated cells (Figure 15). Concomitant with these changes in the cytosol, the mitochondrial levels of cytochrome c decreased significantly. Similar results were also observed when 5 mM glutamate was used (data not shown). Exposure of cortical cells to 1 mM glutamate and 1 μM 17β-E_2 _or Δ^8^, 17β-E_2 _for 6 hours resulted in a significant decrease in the release of cytochrome c into the cytosol (Figure 16). Thus, the levels of cytochrome c in the cytosol from estrogen-treated cells were 30 to 50% lower than the corresponding levels in the glutamate alone treated cells (Figure 16).

## Discussion

In the present study, we used primary fetal rat cortical cell cultures to demonstrate that glutamate can induce neuronal cell death by apoptotic mechanisms and that the process can be reversed or inhibited by equine estrogens such as 17β-E_2 _and Δ^8^, 17β-E_2_. Our results further indicate that glutamate-induced cell death appears to result, to some extent, by a mechanism that involves DNA fragmentation and morphological changes characteristic of apoptosis. These changes are similar to those we and others have reported previously with other neuronal cell models [7,13]. Unlike the mouse hippocampal cell line HT22 [13], the primary cultures of rat cortical cells after day two in culture appear to undergo some degree of apoptotic cell death in absence of glutamate (Figure 2), however, the extent of DNA fragmentation is significantly enhanced after treatment with glutamate. Moreover, compared to the HT22 cells, the primary rat cortical cells appear to be resistant and require higher concentration of glutamate to induce apoptosis. Similar differences were also observed between HT22 cells and PC12-neuronal cells derived from rat adrenal pheochromocytoma [31]. The glutamate induced DNA fragmentation and subsequent cell death was associated with characteristic morphological changes also noted previously in HT22 cells following treatment with β-amyloid and glutamate [13,37]. The results from the present study indicate that these morphological changes associated with apoptosis were prevented by 17β-E_2 _and Δ^8^, 17β-E_2 _(Figure 3). To quantitatively estimate the extent of cell death induced by glutamate, we measured cell viability by MTS assay and cell death by LDH activity released in the media during culture. Results from the MTS assay clearly indicated that cell viability was dependent on the dose of glutamate (Figure 1). Thus, increasing concentrations of glutamate resulted in a dose-dependent decrease in cell viability. Because in the MTS proliferation assay, cells cannot be reused, subsequent quantitative experiments were carried out using LDH release assay. The LDH assay had previously been validated using the MTS assay [29,31].

In the present study, we further observed that glutamate induces cell death of primary rat cortical cells and involves changes in caspase-3 protease. Exposure to glutamate up-regulated caspase-3 protein levels while caspase inhibitors blocked this apoptotic process. These observations indicated that cell death induced by glutamate in primary cortical neurons was via apoptosis.

Caspase-3 was one of the cysteine proteases that played an essential role in apoptosis by cleaving several key cellular proteins such as poly (ADP-ribose) polymerase (PARP), sterol-regulatory element-binding protein (SREBPs), PKC, DNA-dependent protein kinase, DNA-fragmentation factor (DFF) and a number of others [16,19,21,24,25,35,36]. Our studies show that up-regulation of caspase-3 expression preceded neuronal cell death, supporting the possibility that glutamate-induced apoptotic cell death was the consequence of up-regulation of caspase-3 gene in cortical neurons. These observations are consistent with up-regulation of precursor caspase-3 in frontal neuronal cortex of subjects with Alzheimer's disease [5]. This enzyme has been proposed to activate death effector molecules resulting in the fragmentation of genomic DNA and was associated with morphological and structural changes characteristic of apoptosis [16,19,21,25,38].

When cells undergo apoptosis, caspase-3 is initially present as pro-enzyme (32 kDa precursor protein) that is subsequently transformed into the active heterodimeric complexes through a cascade of proteolytic events [14,18]. The active form of caspase-3 is composed of two subunits of Mr 20 kDa (p20) and 11 kDa (p11), which are derived from proteolytic processing of the 32 kDa precursor during apoptosis [21,24,25]. In our study, these p 20 and p11 forms of caspase-3 were not detected in glutamate-induced cell death by immunoblotting analysis with three different kinds of antibodies: **i. **a polyclonal goat antibody caspase-3 p11 (K-19) against the p11 fragment of caspase-3, **ii. **a polyclonal rabbit antibody against caspase-3 (H-227) and **iii. **a monoclonal mouse antibody (E-8) (data not shown with this latter antibody). These observations suggest that either our antibodies are incapable of detecting these fragments or that cells may clear them rapidly as been observed in other studies [39]. Since we observed that glutamate-induced cell death was effectively blocked by both caspase-3 specific inhibitor z-DEVD and pan-caspase-inhibitor z-VAD, these data further provide evidence that caspase-3 protease is not only up-regulated, but is also activated during glutamate-induced cell death. That activation of pro-caspase-3 does indeed occur in glutamate-induced apoptotic cell death of primary rat cortical cells was confirmed by the demonstration that PKC, an endogenenous substrate of active caspase-3, was cleaved into two 45 kDa and 48 kDa BDPs [35,36]. The formation of these BDPs is characteristic of caspase-3 like protease activity [35,36]. The formation of the two BDPs was inhibited by caspase-3 specific inibitor z-DEVD and pan-caspase-inhibitor z-VAD). These results strongly suggest that proleolytic cleavage of PKC involves active caspase-3. These observations were further supported by the direct immunodetection of activated caspase-3 in the primary cultures of rat cortical cells treated with glutamate (Figure 12). The primary antibody was raised against amino acids 163 to 175 of murine caspase-3 and this neo-epitope is present on the p18 subunit of cleaved caspase-3. This antibody recognizes the p18 subunit but not the inactive pro-caspase-3 [40]. Taken together these data strongly indicate that in our neuronal cell cultures, glutamate induces apoptosis that involves active caspase-3. In contrast, it has been suggested that glutamate-induced cell death in HT22 mouse hippocampal cells appears to occur by apoptotic mechanisms that are independent of caspase-3 [13]. Since in HT22 cells, glutamate also induced DNA laddering, mitochondrial proteins such as apoptosis inducing factor (AIF) and endonuclease G (Endo G) released in response to death signals may also play a role in the HT22 neuronal cell line [13]. Whether similar caspase-3 independent pathways are also involved in glutamate-induced apoptosis in primary cultures of rat cortical cells remains to be investigated.

Several signaling pathways are implicated in the initiation of the caspase-3 cascade; one of the well defined pathways for activation of apoptosis is Fas receptor-mediated pathway [41-43]. Fas is a cell surface antigen and a member of tumor necrosis factor (TNF) receptor family [44]. Activation of Fas by its ligand or an agonistic anti-Fas antibody can transmit apoptotic signal by activation of caspase-3 and induces apoptosis in T-lymphocytes and malignant cells [26,45,46]. In vitro, cross-linking of Fas antibody to its antigen increases caspase-3 activity and induces apoptotic cell death in Jurkat cells [47]. However, in primary cortical cells, we did not observe any change in Fas receptor protein levels following glutamate treatment. These results suggest that activation of caspase-3 in neuronal cortical cells and their apoptotic demise induced by glutamate is independent of Fas receptor mediated pathway. However, the Fas mediated pathway is complex and further studies are required to establish whether this pathway is or not involved in glutamate-induced cell death in cortical cells. In contrast to glutamate, chronic morphine administration has been shown to induce up-regulation of Fas receptor and caspase-3 precursor protein in the rat neuronal cells [48,49]. Thus, whether Fas receptor mediated pathway plays a role in up-regulation of caspase protein or caspase-3 activation and apoptotic cell death may depend on the nature of neurotoxic agents being used.

Another recently characterized mechanism for pro-caspase-3 activation involves translocation of the respiratory chain protein, cytochrome c (Apaf-2), from mitochondria to the cytosol during the induction of apoptosis by a variety of different agents in non-neuronal and neuronal cells such as cerebellar granule cells [21,50]. Cytosolic cytochrome c binds to Apaf-1 in the presence of dATP and this leads to the recruitment and activation of caspase-9 and subsequent activation of caspase-3 [23,25]. In mitochondria cytochrome c resides in the inter-membrane space and matrix as a soluble protein and it functions as an electron carrier in oxidative phosphorylation [21]. The mechanism responsible for the translocation of cytochrome c from mitochondria into cytosol is not known. Cytochrome c release can occur upon interaction of pro-apoptotic protein with the outer mitochondria membrane protein such as Bax [51,52] or can be induced by interaction with elevated calcium or reactive oxygen species (ROS) with mitochondria. This results in mitochondrial dysfunction and a reduction in mitochondrial trans-membrane potential [53-55]. Our present studies demonstrate that glutamate-induced cell death in primary cortical cells is accompanied by the early release of cytochrome c into the cytoplasm that precedes changes in caspase-3 protease. Although we were unable to directly detect by Western blot analysis, the active caspase-3 fragments p20 and p11, the presence of active caspase-3 was confirmed as discussed above. Since there was a close correlation between the time course of cytochrome c release from the mitochrondria and changes in caspase-3 protease levels; it provides a possible mechanism whereby caspase-3 protease is not only up-regulated, but also activated by cytochrome c during glutamate-induced apoptotic cell death of primary cortical cells. Whether cytochrome c release from the mitochondria to cytosol is involved in the regulation of caspase-3, remains to be investigated.

We have previously reported that a number of equine estrogens, which are components of the drug CEE (conjugated equine estrogen) used extensively for management of vasomotor symptoms and osteoporosis in postmenopausal women, are potent antioxidants and protect neuronal cells against cell death induced by oxidized LDL and glutamate [28,29,56,57]. In keeping with these observations, the results from the present study indicate that in primary cortical cells, these equine estrogens prevent cell death by reducing glutamate-induced cytochrome c release from mitochondria and caspase-3 protein levels. To our knowledge, this is the first study that demonstrates that equine estrogens can prevent glutamate-induced translocation of cytochrome c from mitochondria to the cytosol in the rat primary cortical cells. Previous studies have shown that 17β-E_2 _induces release of cytochrome c from heart mitochondria [58] and also decreases cytosolic cytochrome c levels in hippocampus following global ischemia [59]. These observations indicate that equine estrogens protect against glutamate-induced apoptosis of primary cortical cells at least in part by inhibiting caspase-dependent apoptotic pathway. However, the mechanism by means of which estrogens reduce cytochrome c release from mitochondria is not yet fully understood. Some have suggested that the mechanism of estrogen-mediated neuroprotection involves regulation of mitochondrial calcium (Ca^2+^) and Bcl-2 expression [59]. Exposure of glutamate has also been associated with an increase of cytosolic Ca^2+ ^in cortical cells [3,53] and up-regulation of pro-apoptotic protein Bax in neuronal cell line, HT22 [13]. Whether these changes induced by glutamate are involved in the mitochondria release of cytochrome c into the cytosol, remains to be investigated.

The cortical neuronal cells used in the present study contain both estrogen receptors ERα and ERβ (Bhavnani and Zhang, unpublished data), and therefore whether the mechanism by means of which estrogens exert their neuroprotective effects remain to be elucidated. In general, the current evidence indicates that there are at least two mechanisms for estrogen action: i) the genomic mechanism mediated via two nuclear receptors ERα and ERβ, and ii) the non-genomic mechanism mediated via putative membrane receptors and these have been recently reviewed [32]. Although the concentrations of estrogens that protect the cortical neurons from glutamate toxicity are pharmacological doses, these levels can however, be potentially attained in a postmenopausal women taking daily 0.625 mg of (CEE) [61]. These estrogen effects are most-likely mediated to some extent by the non-genomic mechanism, and may be related to the estrogens' antioxidant property [28,29]. This is in keeping with our previous observations that although 17β-E_2 _has higher affinity for ERα and ERβ, it is possible that some of these effects are due to the antioxidant properties of estrogens. However, we have also demonstrated [33] that Δ^8^, 17β-E_2 _expresses its biological effects to a two fold greater extent via ERβ than 17β-E_2 _[33]. Since the localization of ERα and ERβ in the brain is differential [[32] and references therein] further detailed studies are needed to elucidate the molecular mechanisms involved in the neuroprotective aspects of estrogens.

Recent studies further report that estrogen receptor (ERβ) is localized in the mitochondrial membrane and estrogen can directly modulate the mitochondrial content of Ca^2+^ and mitochondrial trans-membrane potential [61,62]. The role of the novel estrogen membrane receptors in apoptotic cell death induced by glutamate remains to be investigated. We along with others, have previously demonstrated that estrogens prevent cell death induced by glutamate [13] or β-amyloid [63] via modulation of Bcl-2 family of proteins. Thus, it is possible that in the neuronal cell model, estrogens decrease glutamate-induced cytochrome c release from mitochondria by protecting mitochondrial trans-membrane potential via binding estrogen receptor on mitochondrial membrane as well as up-regulation of anti-apoptotic protein Bcl-2. Studies to further delineate these mechanisms are being initiated.

## Conclusion

In the primary cortical cells, glutamate-induced apoptosis is accompanied by up-regulation of caspase-3 that can be blocked by caspase protease inhibitors. These effects of glutamate on caspase-3 appear to be independent of changes in Fas receptor, but are associated with the rapid release of mitochondrial cytochrome c, which precedes changes of caspase-3 protein levels leading to apoptotic cell death. This process was differentially inhibited by estrogens with the novel equine estrogen Δ^8^, 17β-E_2 _being more potent than 17β-E_2_, To our knowledge, this is the first study to demonstrate that equine estrogens can prevent glutamate-induced translocation of cytochrome c from mitochondria to cytosol in rat primary cortical cells.

## Methods

### Materials

All primary and secondary antibodies and other reagents were purchased from commercial sources: rabbit polyclonal antibody to caspase-3 (H-227: *sc 7148*) and goat polyclonal antibody to caspase-3 p11 (K-19: *sc-1224*). Both of these antibodies react specifically with precursor protein: Mr 32 kDa and the active caspase-3: Mr 20 (p 20) and 11 kDa (p 11) fragments. Rabbit polyclonal antibody to protein kinase c (P4334) (PKC, crossreacts with PKCα /and PKC δ) purchased from Sigma. Goat polyclonal antibody to Actin (I-19: *sc1616*) (all from Santa Cruz, California), mouse monoclonal antibody to Fas raised against Fas extracellular domain (CD95:*AF435*) (R&D System, Inc. Minnepolis, MN), mouse monoclonal antibody cytochrome c (7H8.2C12) (BD PharMingen, Mississauga, ON). Secondary antibodies were obtained from Sigma (Saint Louis, Missouri). SuperSignal^® ^West Pico Chemiluminescent Substrate (Pierce, Rockford, IL). Z-Val-Ala-DL-Asp (OMe)-fluoromethylketone (z-VAD-fmk) and z-Asp (OMe)-Glu (OMe)-Val-DL-Asp (OMe)- fluoromethylketone (z-DEVD-fmk) (Bachem Bioscience Inc., King of Prussia, PA) and the Cytotox 96 Non-radioactive Cytotoxicity Assay Kit (Promega G1780, from VWR, Toronto, Ontario, Canada). Neurobasal, B27, Hanks' buffered salt solution (HBSS) and antibiotics were from Gibco Life technologies (Burlington, Ontario, Canada). Active Caspase-3 Detection FITC kit # 280000 was obtained from Stratagene (La Jolla, CA).

### Animals

Timed pregnant Sprague-Dawley rats were obtained from commercial sources. All procedures were reviewed and approved by the Institutional Animal Care and Use Committee at St. Michael's Hospital, University of Toronto, in accordance with the guidelines of the Canadian Council on Animal Care.

### Cell culture

Eighteen to twenty day old pregnant Sprague-Dawley rats were sacrificed by cervical dislocation. The fetuses were delivered and fetal skulls were carefully opened and the meninges were removed and the brain processed essentially as described by Brewer et al. [64]. Briefly, two pieces of cortex (0.5–1.5 × 1–2 mm) were cut from the posterior dorsal surface and dissociated in HBSS (Ca^2+ ^and Mg^2+ ^free). Following dissociation, cells were suspended in 2 X volume of HBSS with Ca^2+ ^and Mg^2+ ^and allowed to settle for 3 to 5 minutes. The supernatant was transferred to new tubes and centrifuged at 1100 rpm (200 g) for 1 min. The pellets were suspended in HBSS buffer. Fresh cell suspensions were plated onto poly-L-lysine-coated 96-well plates or 6-well plates containing neurobasal medium supplemented with 2% B27, 0.5 mM L-glutamine, 25 μM glutamic acid, 120 mg/L penicillin and 260 mg/L streptomycin and incubated at 37 C in a humidified incubator with 5% CO_2_/95% air. Culture medium was replaced every other day. Experiments were performed on days 7 to 8 of cultures in neurobasal medium containing 2% antioxidant free B27 supplement. Under these serum-free culture conditions, only neuronal cells survive.

### Determination of glutamate-Induced cell cytotoxicity

During the development of this work, the lactate dehydrogenase (LDH) cytotoxicity assay (Promega G1780, VWR Toronto, Ontario, Canada) and the Cell Titer 96^® ^Aqueous Non-radioactive cell proliferation assay (MTS assay, Promega G5430) were used to assess cell death and cell viability respectively [31]. In MTS (3-[4,5-dimethylthiazol-2 yl]-5 [3-carboxymethoxyphenyl]-2H-tetrazolium, inner salt) the formation of a formazan product occurs only in live cells, however, once treated with MTS, the cells are not useable for other measurements. We have previously demonstrated [31] a strong correlation between the MTS and LDH assays [31]. Since we wanted to use the cells for various determinations, the LDH assay for cell death was selected as in this assay only the supernatant is used and the cells can be used for other measurements. Thus after the initial determination of the glutamate dose response curve, only the LDH assay was used in all subsequent experiments. Cell death was measured by the Cytotox 96 Assay kit, which quantitatively measures the release of lactate dehydrogenase into the medium following cell lysis or cell death as described previously [13]. In all experiments, cultures were also treated with 0.1% Triton X-100 to lyse the cells, and LDH levels measured under these conditions, were taken as the maximal LDH release (100% cell death). The results were expressed as a percentage of maximum LDH release.

### Assessment of apoptosis by DNA fragmentation

Apoptotic cells produce characteristic DNA ladders made up of nucleotide fragments which are visualized by staining with ethidium bromide following DNA-agarose gel electrophoresis. We used DNA fragmentation as one of the criteria for apoptotic cell death and this was determined as described previously [13]. Briefly, primary cortical cells isolated from 18 to 20 day old fetal rat brains were cultured for up to 7 days and then treated with 1 mM glutamate for 18 h. The cell monolayers were washed with ice cold TBS (20 mM Tris-HCl, pH 7.6, 137 mM NaCl) and then DNA was isolated as described previously [13]. The DNA (5 μg) was electrophoresed on 1.5% agarose gel for 1.5 h at 100 V. The DNA fragments were visualized by staining with ethidium bromide and detected under UV transillumination.

### Western blot analysis

Protein levels of Caspase-3, Fas or PKC were determined by Western blotting using a polyclonal and monoclonal antibody raised against caspase-3 or Fas or PKC as described previously [13]. In brief, cells were washed twice with cold PBS, harvested using a cell scraper, and lysed in buffer (9 mM Na_2_PO_4_, 1.7 mM NaHPO_4 _150 mM NaCl, pH 7.4), containing 1% Nonidet P-40 (Sigma, Toronto, Canada), 0.5% sodium deoxycholate, 0.1% SDS and 1 mM phenyl methyl sulfonyl fluoride) for 20 min on ice. Cell lysates were centrifuged at 10,000 g at 4 C for 10 min. The protein concentration was determined by Bradford method (BioRad, Toronto, Canada). Cell lysates containing 10 to 20 μg protein were added in equal volume of 2 X reducing sample buffer (100 mM Tris-CI pH 6.8, 200 mM dithiothretiol, 4% SDS, 20% glycerol, and 0.2% Bromophenol blue) and heated at 100 C for 3 min. The samples were electrophoresed on discontinuous 10% polyacrylamide gel electrophoresis under constant current (14–15 mA). Separated proteins were electrotransfered onto a Protan nitrocellulose membrane (Schleicher & Schuell Inc., Keene N.H). The blots were blocked with 5% non-fat milk in TBST (20 mM Tris-HCl pH 7.6, 137 mM NaCl, 0.05% Tween-20) at 4 C overnight and then incubated with primary antibody for 1 to 2 h at room temperature, washed three times with TBST and incubated (1–2 h) with appropriate horse radish peroxidase (HRP)-conjugated second antibody. The membranes were washed three times with TBST and the immunoblots were visualized on X-ray films after exposure to enhanced chemiluminescence reagent (ECL) (Amersham, Toronto, Canada). Actin bands were monitored on the same blot to verify consistency of protein loading. Briefly, the Immunoblots were stripped with TBST containing 0.04% sodium azide for 30 min at room temperature. The blots were probed with anti-actin primary antibody and second antibody (anti-goat) as described above. The molecular size of protein was determined by running pre-stained protein markers in an adjacent lane, omitting primary antibody as a procedural control. The band intensity was measured from scanned images using UN-SCAN IT Gel Automated Digitizing system, Version 5.1 (Silk scientific, Inc, Orem, Utah, USA), software.

### Statistics

Each experiment was repeated at least three times and combined data were compared using the Student's paired *t- *test. Analysis of variance (ANOVA) when appropriate, was used for multiple comparisons. Significance was set at P < 0.05.

### Measurement of cytochrome c translocation

The cell homogenates, mitochondria and cytosolic fractions were prepared essentially as described by Atlante et al. [54]. The cells were washed twice with ice-cold PBS, pH 7.4 and then suspended in cold isotonic buffer (250 mM sucrose, 20 mM Hepes-KOH, 10 mM KCI, 1.5 mM MgCI_2_, 1 mM Na-EDTA, 1 mM Na-EGTA, 1 mM dithiothretiol and protease inhibitors-complete cocktail). After 15 min incubation on ice, cells were homogenized using a glass homogenizer (25 stokes) at 4 C. Cell homogenates were spun at 750 g for 10 minutes and the pellets containing the nuclei and unbroken cells were discarded. The supernatant was then centrifuged at 10,000 g for 15 minutes. The pellets containing mitochondria was stored at -80 C until processed. The 10,000 supernatant was further centrifuged at 100,000 g for 1 hour at 4 C to obtain the cytosolic fraction (S-100). The intactness of the mitochondrial membranes was checked by assaying mitochondrial specific enzyme glutamate dehydrogenase in mitochondria, mitochondria treated with Triton X-100 and in cytosol (S-100) as described previously [54]. GDH was only detectable in mitochondria and mitochondria treated with Triton X-100. It was undetectable in cytosol. Thus, the cytosol fraction was free of mitochondrial contamination. Following analysis of protein concentration, the levels of cytochrome c in these two fractions were analyzed by Western blot on 12% SDS-polyacrylamide gel. Cytochrome c was detected using murine anti-cytochrome c antibody as described above.

### Assessment of the effects of caspase inhibitors

Caspase inhibitors were used as described previously [65] caspase-3-inhibitor z-DEVD-fmk and a pan-caspase-inhibitor z-VAD-fmk. The concentrations of inhibitors tested were 25, 50 and 100 μM. Both were prepared as 20 mM stocks in DMSO and stored at -80 C. Primary cultures were incubated with the inhibitors for 30 min prior to the addition of glutamate. Effects of inhibitors on glutamate-induced cell death were measured with LDH release assay as described above.

### Detection of active caspase-3 by immunofluorescence

The primary cortical cells were cultured for 7 days in 96-well microplates and then treated with or without glutamate for 3 h and 6 h. Cells were then processed essentially as described by the manufacturer of the assay kit (Stratagene). Briefly, the microplates were centrifuged (200 × g) for 2 minutes and the supernatant discarded. The cells were fixed for 15 minutes following which the fixative was aspirated and the cells washed twice with phosphate buffered saline (PBS) containing 1% saponin. The cells were then incubated with the primary anti-cleaved caspase-3 antibody in PBS + 1% saponin for 30 minutes at room temperature. The cells were then rewashed and incubated for 30 minutes with secondary FITC-goat anti-rabbit antibody and then washed twice and the relative fluorescence measured using a microplate reader (excitation 490 nm; emission 520 nm).

## Authors' contributions

***YMZ ***is a postdoctoral fellow who participated in the development of the hypothesis, study design and carried out most of the experimental work and preparation of the manuscript.

***BB ***conceived the study and participated in the development of the hypothesis, the study design, and overall direction of the study and preparation of the manuscript.
